# Outcome of burr-hole aspiration of brain abscess

**DOI:** 10.12669/pjms.335.13022

**Published:** 2017

**Authors:** Shakeel Ahmad, Lal Rehman, Ali Afzal, Raza Rizvi

**Affiliations:** 1 Dr. Shakeel Ahmed, MBBS. Department of Neurosurgery, Jinnah Postgraduate Medical Center, Karachi, Pakistan; 2 Dr. Lal Rehman, FCPS. Department of Neurosurgery, Jinnah Postgraduate Medical Center, Karachi, Pakistan; 3 Dr. Ali Afzal, MBBS. Department of Neurosurgery, Jinnah Postgraduate Medical Center, Karachi, Pakistan; 4 Dr. Raza Rizvi, MS. Department of Neurosurgery, Jinnah Postgraduate Medical Center, Karachi, Pakistan

**Keywords:** Brain abscess, Burr-hole aspiration

## Abstract

**Objective:**

To determine the clinical outcome of burr-hole aspiration of brain abscess.

**Methods:**

We analyzed 100 cases of intracranial abscess, treated surgically from January 2015 and October 2016 at Jinnah Postgraduate Medical Centre (JPMC). All patients were treated with burr hole aspiration. Medical records were analyzed for demographics, clinical presentation, predisposing factors, abscess location on imaging and clinical outcomes were charted.

**Results:**

The study included 100 patients with 73 (73%) males and 27 (27%) females with a mean age of 36.69±10.96 years. Mean duration of signs and symptoms was 8.50±4.2 days. The most common presenting complaint was altered sensorium in 70 (70%) patients and commonest source of infection was otitis media seen in 27 patients (27%). The GCS on presentation was 13 in 57 (57%) cases. The parietal region was the most common site in 43 patients (43%), followed by frontal region in 33 patients (33%). Complete resolution of abscess with recovery of preoperative neuro-deficit was seen in 77 (77%) patients and recovery with major neuro-deficit was observed in 10 (10%) cases while 13 (13%) patients expired.

**Conclusion:**

Early diagnosis, optimum follow-up and timely burr-hole aspiration are the keys in the proper management of brain abscess.

## INTRODUCTION

Brain abscess is infrequent but potentially life-threatening infection. It involves a focal, intraparenchymal collection of pus^1^ seeded by septic foci in contiguous or distant region(s). The pathogenesis of intracranial abscess requires inoculation of a microorganism into the brain parenchyma in an area of devitalized brain tissue or with poor microcirculation. The lesion evolves from early cerebritis to the stage of organization and capsule formation which is well-vascularized.^2^,^3^ The mode of entry of organisms could be by contiguous spread, hematogenous dissemination, or following trauma.^4^

Brain abscess is more common in developing countries with an incidence of 8% compared to 1-2% in Western countries and poses a public health challenge.^5^ Despite the advent of modern neurosurgical techniques, new antibiotics and non-invasive neuroimaging guided procedures that have revolutionized its treatment, it still poses therapeutic challenge.^6^,^7^ Infections in contiguous structures are responsible for brain abscess in 40-50% of cases. Other predisposing factors include cyanotic heart diseases and direct inoculation of bacteria into the brain during surgery or head injuries. In up to 25% of cases, there may be no clear predisposing factors.^8^

The common presenting features are headache, vomiting, fits, fever, focal deficits and impaired consciousness.^9^,^10^ The most frequent intracranial locations are: frontotemporal, frontoparietal, parietal, cerebellar, and occipital lobes.^11^ Treatment may be conservative or surgical which depends upon stage, size, location and number of abscesses and clinical status of the patient.^12^ The purpose of this study was to find out various presenting complaints and sites of brain abscesses and determination of clinical outcome of its management.

## METHODS

The study was conducted in the department of neurosurgery at JPMC, Karachi from January 2015 to October 2016, with Institutional Review Board (IRB) approval. One hundred patients were included in this study with ages between 13 to 65 years. Patients with deep-seated or infratentorial abscess, multiple and multiloculated abscesses, recurrent abscess and patients ≤ 12 years old were excluded from the study. Deep seated lesions were managed conservatively if small or aspirated stereotactically.

All patients underwent burr-hole aspiration followed by intravenous antibiotics regimen. Significant residual abscess as greater than 50% of original volume (assessed pre operatively) found on postoperative imaging, were reaspirated. Patients were kept on empirical antibiotic regimen till availability of culture reports that was continued after discharge for a total of 12 weeks with six weeks intravenous and six weeks oral therapy. In all of our patients, we have given steroids which were tapered over two weeks. Anti-epileptics were given to all our patients with frontal and temporoparietal abscesses.

Data was collected on the participant’s age, sex, presenting complaints, duration of sign & symptoms, various predisposing factors, location of brain abscess on imaging (CT scan/MRI brain) & outcomes of brain abscess in terms of clinical improvement and mortality. Repeat CT was performed on first postoperative day, one week after surgery and at three months to assess the residual volume of the abscess. Patients were followed up for total of three months at one week, fortnightly for 1^st^ month and then at three months. All information was recorded in proformas.

Data was analyzed using SPSS version 21. All the data were expressed as mean ± SD (standard deviation) and percentage (%), as appropriate. The statistical significance of differences between the values was assessed by Chi square test and p value of < 0.05 was considered statistically significant.

## RESULTS

**Fig.1:**
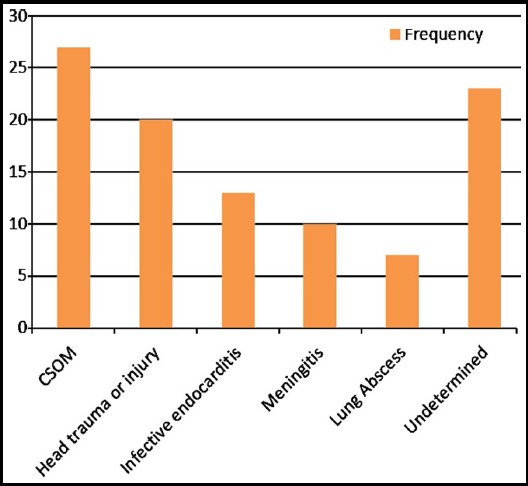
Etiology.

Among 100 patients, 73 (73%) patients were male and 27 (27%) were female. The ages varied from 15 to 63 years with a mean of 36.69 ±10.96 years. Altered sensorium was the most common presentation in 70 (70%) patients, followed by headache in 63(63%) (Table-I). The duration of symptoms varied from5 to 15 days with a mean of 8.50±4.2 days. There was a predisposing factor (77%), in 27patients (27%), the source of infection was chronic otitis media while twenty (20%) had history of trauma or head surgery, 13 patients (13%) had infective endocarditis [Fig.1]. In 23 patients (23%), the source could not be determined. In 57 (57%) patients GCS was 13/15, followed by 10/15 in 20 patients (20%).

**Table-I:** Presenting Complaints.

		*Frequency*	*Percent*	*Valid Percent*	*Cumulative Percent*
Altered Sensorium	Yes	70	70.0	70.0	70.0
	No	30	30.0	30.0	100.0
	Total	100	100.0	100.0	
Fever	Yes	60	60.0	60.0	60.0
	No	40	40.0	40.0	100.0
	Total	100	100.0	100.0	
Headache	Yes	63	63	63	63
	No	37	36.7	36.7	100.0
	Total	100	100.0	100.0	
Vomiting	Yes	37	36.7	37	37
	No	63	63.3	63.3	100.0
	Total	100	100.0	100.0	
Focal Neurological Deficit	Yes	30	30.0	30.0	30.0
	No	70	70.0	70.0	100.0
	Total	100	100.0	100.0	
Seizures	Yes	40	40.0	40.0	40.0
	No	60	60.0	60.0	100.0
	Total	100	100.0	100.0	

The commonest location for abscess was the parietal region in 44 patients (44%) followed by frontal in 33(33%), temporal 13 (13%), and occipital regions in 10 (10%). Forty patients (40%) had no residual abscess on post operative CT while 17(17%) patients had significant residual abscesses that were re-aspirated. The remaining 43 patients (43%) had minimal residual abscesses and were managed conservatively (Table-II). Complete resolution of abscess with recovery of preoperative neuro-deficit was seen in 77 (77%) patients and recovery with majorneuro-deficit was observed in 10 (10%) cases and13 (13%) patients expired (Table-III).

**Table-II:** Findings at repeat CT scan within one week after surgery.

	*Frequency*	*Percent*	*Valid Percent*	*Cumulative Percent*	*P-value*
No residual abscess	40	40.0	40.0	40.0	0.700
Significant residual abscess	17	17	17	17	
Minimal residual abscess	43	43	43	43	
Total	100	100.0	100.0	100.0	

**Table-III:** Outcome of brain abscess.

	*Frequency*	*Percent*	*Valid Percent*	*Cumulative Percent*	*P-value*
Death	13	13	13	13	0.573
Complete resolution of abscess with complete recovery of neuro-deficit	77	77	77	90.0	
Recovery with major neuro-deficit	10	10.0	10.0	100.0	
Total	100	100.0	100.0		

## DISCUSSION

Brain abscess presents with symptoms and signs depending upon its localized and generalized effects. In Gadgil et al. study, presenting complaints at the time of presentation included headache in 14 patients (42%), nausea or vomiting in six (18%), altered mental status in 11 (33%), visual complaints in five (15%), and seizures in three (9%).^13^ While in Tan WM et al.^14^ study altered sensorium was noted to be occurring in most of the brain abscess patients (82.4%) followed by fever (66.7%), headache (62.7%), vomiting (39.2%) and focal neurological deficit (33.3%) similar to our findings of altered sensorium in 70(70%) followed by headache 63 (63%), fever 60(60%), vomiting 37 (37%), seizures in 40 (40%) and focal neurological deficit in 30 (30%) patients.

The common predisposing factors of a brain abscess are Chronic suppurative otitis media (CSOM), congenital cyanotic heart disease, and paranasal sinusitis.^15^,^16^ In our study in the majority of cases, there was a predisposing factor (76.7%). In 27 patients (27%), the source of infection was from ear infection, 20 patients (20%) had history of trauma and/or head surgery; 10 patients (10%) had meningitis; 13 patients (13%) had infective endocarditis and two patients (7%) had lung abscess. In 23 patients (23%) of cases, the source was not determined. This was consistent with findings of Gadgil et al. where, in the majority of cases, there was a predisposing factor (80.4%). In sixteen patients (31.4%), the source of infection was from the heart, either due to cyanotic heart disease or infective endocarditis. Eleven patients (21.6%) had history of trauma and/or head surgery; four patients (7.8%) had meningitis; five patients (9.8%) had ear infection (three with mastoiditis) and three patients (5.9%) had lung abscess. In 19.6% of cases the source was not determined.^13^

Immunosuppression due to disease or therapy is emerging as an important risk factor for the development of brain abscess.^12^ The use of steroids in brain abscess is controversial. Some experts believe steroid therapy can reduce antibiotic penetration into the abscess or increase the risk of ventricular rupture. However, in patients with severe cerebral edema, a short-course of steroids may be beneficial.^17^,^18^ We gave steroids to all our patients and it was tapered over two weeks. Antiepileptics were given in all patients with frontal and temporoparietal abscesses as seizure is a long term risk for upto 30-50% of patients suffering from brain abscess.^19^

Brain abscess can occur anywhere in the brain for which CT and MRI brain are the diagnostic tools to confirm its exact location. The most common location for the abscess was the frontal lobe in 24 patients (47.1%) followed by parietal (29.4%), temporal (13.7%), and occipital regions (9.8%) as similar to findings stated by Brook.^7^ In Tan WM et al. study, on repeat CT scan within one week after surgery, 22(43.1%) patients had no residual abscess. There were six patients (11.8%) with significant residual abscesses whereas the remaining 23 patients (45.1%) had minimal residual abscesses.^14^

In addition to antibiotic therapy, early surgical intervention fastens recovery provides early improvement in clinical symptoms. The procedure involves performing an initial craniotomy followed by drainage or aspiration of the contents of the abscess or surgical excision. However, inadequate aspiration or drainage is a common failure for deep-seated brain abscesses. The role of image guided surgery is now on the rise. Minimally invasive image-guided keyhole aspiration of cerebral abscesses using per operative neuronavigation with CT or MR guidance are the new alternatives or using stereotaxy. The advantage is decreased intraoperative blood loss, operative duration, and length of incision.

In a study by Meng et al.^20^, statistical difference was observed in above parameters in patients undergoing key hole aspiration versus formal surgery but no differences were observed in duration of hospital stay and antibiotic therapy and postoperative neurological recovery time were all increased in the aspiration group. A case series by Boviatsis^21^ was done on 12 patients who underwent drainage of intracerebral abscesses under stereotactic guidance. Ten patients had solitary lesions and two had multiple abscesses. The study reported improvement in all patients without any mortality or postoperative complication. A second aspiration was required in only one patient due to recurrence. Stereotaxy is beneficial not only for deep seated lesions or lesions in eloquent cortex but helpful diagnostic tool where diagnosis is in doubt.

In our study, complete resolution of abscess with recovery of preoperative neuro-deficit was seen in 77 (77%) patients and recovery with major neuro-deficit was observed in 10 (10%). Mortality in our study was 13% which is comparable to Chowdhury et al. study^22^, where mortality was 22(13.6%) cases. Complete resolution of abscess with complete recovery of preoperative neuro-deficit was seen in 80.86% cases and recovery with major neuro-deficit was observed in 5.5% cases.

### Limitations of the study

It is a single center study with short follow up. Larger trials with comparison of newer image guided procedures are needed but burr hole aspiration remains a viable option especially in emergent settings and in centers where latest imaging facilities are not available.

## CONCLUSION

Brain abscess can lead to death of the patient if it is not managed timely. It has excellent results if it is operated in time with proper cover of antibiotics and regular follow-up.

### Authors` Contribution

**LR** conceived and designed the study.

**SA** did statistical analysis and editing of manuscript.

**AA** did data collection and manuscript writing.

**RR** did review and final approval of manuscript.

## References

[1] Osenbach RK, Zeidman SM (1999). Pyogenic Brain Abscess. Infections in Neurological Surgery, Diagnosis and Management.

[2] Gortvai P, De Louvois J, Hurley R (1987). The bacteriology and chemotheraphy of acute pyogenic brain abscess. Br J Neurosurg.

[3] Khattak A, Rehman RU, Anayatullah Alam W (2010). Etiological factors of brain abscess. Pak J Med Sci.

[4] Zhang C, Hu L, Wu X, Hu G, Ding X, Lu Y (2014). A retrospective study on the aetiology, management, and outcome of brain abscess in an 11-year, single-centre study from China. BMC Infect Dis.

[5] Khaja M, Adler D, Lominadze G (2017). Expressive aphasia caused by Streptococcus intermedius brain abscess in an immunocompetent patient. Int Med Case Rep J.

[6] Wilson HL, Kennedy KJ (2013). Scedosporiumapiospermum brain abscesses in an immunocompetent man with silicosis. Med Mycol Case Rep.

[7] Ansari MK, Jha S (2014). Tuberculous brain abscess in an immunocompetent adolescent. J Nat Sci Biol Med.

[8] Carpenter J, Stapleton S, Holliman R (2007). Brain abscess. Eur J Clin Microbiol Infec Dis.

[9] Yang KY, Chang WN, Ho JT (2006). Postneurosurgical nosocomial bacterial brain abscess in adults. Infection.

[10] Tunkel AR, Wispelwey B, Scheld WM, Mandell GL, Bennett JE, Dolin R (2010). Brain abscess. Principles and practice of infectious diseases.

[11] Brook I (2017). Microbiology and treatment of brain abscess. J Clin Neurosci.

[12] Mylonas Al, Tzerbos FH, Mihalaki M, Rologis D, Boutsikakis I (2007). J Craniomaxillofac Surg.

[13] Gadgil N, Patel A, Gopinath S (2013). Open craniotomy for brain abscess:A forgotten experience?. SurgNeurol Int.

[14] Tan WM, Adnan JS, Haspani MS (2010). Treatment outcome of superficial cerebral abscess:An analysis of two surgical methods. Malays J Med Sci.

[15] Takeshita M, Kagawa M, Yonetani H, Izawa M, Yato S, Nakanishi T (1992). Risk factors for brain abscess in patients with congenital cyanotic heart disease. Neurol Med Chir (Tokyo).

[16] Loeffler JM, Bodmer T, Zimmerli W, Leib SL (2001). Nocardial brain abscess:observation of treatment strategies and outcome in Switzerland from 1992 to 1999. Infection.

[17] Tseng JH, Tseng MY (2006). Brain abscess in 142 patients:factors influencing outcome and mortality. Surg Neurol.

[18] Benninger F, Steiner I (2013). Steroids in bacterial meningitis:Yes. J Neural Transm.

[19] Osenbach RK, Loftus CM (1992). Diagnosis and management of brain abscess. Neurosurg Clin N Am.

[20] Meng X-H, Feng S-Y, Chen X-L, Li C, Zhang J, Zhou T (2015). Minimally invasive image-guided keyhole aspiration of cerebral abscesses. Int J Clin Exp Med.

[21] Boviatsis EJ, Kouyialis AT, Stranjalis G, Korfias S, Sakas DE (2003). CT-guided stereotactic aspiration of brain abscesses. Neurosurg Rev.

[22] Chowdhury FH, Haque MR, Sarkar MH, Chowdhury SN, Hossain Z, Ranjan S (2015). Brain abscess:Surgical experiences of 162 cases. Neuroimmunol Neuroinflamm.

